# Brazilian Protocol for Sexually Transmitted Infections 2020: Zika virus infection

**DOI:** 10.1590/0037-8682-609-2020

**Published:** 2021-05-17

**Authors:** Geraldo Duarte, Angélica Espinosa Miranda, Ximena Pamela Diaz Bermudez, Valeria Saraceni, Flor Ernestina Martinez-Espinosa

**Affiliations:** 1 Universidade de São Paulo, Faculdade de Medicina de Ribeirão Preto, Ribeirão Preto, SP, Brasil.; 2 Ministério da Saúde, Secretaria de Vigilância em Saúde, Brasília, DF, Brasil.; 3 Universidade de Brasília, Programa de Pós-Graduação em Saúde Coletiva, Brasília, DF, Brasil.; 4 Secretaria Municipal de Saúde do Rio de Janeiro, RJ, Brasil.; 5 Fundação Oswaldo Cruz, Instituto Leônidas e Maria Deane, Manaus, AM, Brasil.

**Keywords:** Zika virus, Sexually transmitted infections, Vertical transmission, Microcephaly, Congenital abnormality, Disease prevention

## Abstract

This article addresses the vector, sexual and vertical transmissions of the Zika virus, a topic covered in the Clinical Protocol and Therapeutic Guidelines for Comprehensive Care for People with Sexually Transmitted Infections, published by the Brazilian Ministry of Health in 2020. Although in Brazil Zika virus is transmitted more predominantly by *Aedes aegypti*, the vertical and sexual transmission routes are of significant importance for reproductive health. Sexual transmission demands specific prophylactic interventions, including the use of male or female condoms, especially among couples in a risk situation and planning pregnancy. Vertical transmission is linked to severe structural abnormalities of the central nervous system, and there is still no vaccine or known pharmacological resources that can prevent it. As the disease is predominantly asymptomatic, failure to comply with the basic principles of care and guidelines associated with the spread of the infection transcends the severity of the disease's symptoms.

## Highlighted excerpt

Although in Brazil Zika virus is predominantly transmitted by the *Aedes aegypti* mosquito, vertical and sexual transmission routes are important for reproductive health. Vertical transmission causes severe central nervous system structural abnormalities.

## FOREWORD

This article addresses the chapter on Zika virus in the Clinical Protocol and Therapeutic Guidelines for Comprehensive Care (CPTG) for People with Sexually Transmitted Infections (STI), published by the Health Surveillance Department of the Brazilian Ministry of Health. For the elaboration of the CPTG, a selection analysis of the evidence available in literature was performed, and a panel of specialists discussed it. The CPTG was approved by the National Committee for the Incorporation of Technologies in the Brazilian National Health System (Conitec)^1^ and updated by the team of specialists in STI in 2020.

## EPIDEMIOLOGICAL ASPECTS

Zika virus belongs to the *Flaviviridae* family, genus *Flavivirus*. It was first isolated in Uganda (Africa) in 1947^2^ ^,^ ^3^. Infections in human beings were sporadic for around half a century before the epidemic outburst in some islands in the Pacific Ocean and South America^4^. During such dissemination period in different mesological conditions, the virus developed important mutations, characterizing two different lineages: the African lineage and the Asian lineage^5^. In parallel to such genomic adaptations, the Zika virus's pathogenic potential has changed^6^ ^,^ ^7^. There is current evidence that viral transmission forms, including the sexual one, are influenced by these viral mutations^8^. 

In April 2015, the Zika virus was first identified in the Americas, with the first case being reported in Bahia, Brazil. By the end of January 2016, its native circulation had already been registered in more than 20 countries or territories in South, Central, and North America, the Caribbean, and Cape Verde, in West Africa^4^. 

**FIGURE 1:**
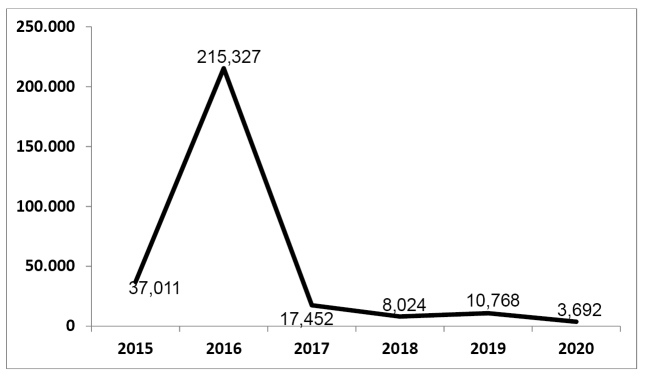
Number of Zika virus infection cases notified in Brazil, 45^th^ Epidemiological Week of 2015 until 23^rd^ Epidemiological Week of 2020.

**Source:** Numeric information taken from references^9^^,^^10^^,^^11^, and^12^.

The evolution of Zika virus infection incidence in Brazil is shown in Figure 1. An important reduction in cases can be seen after the 2015-2016 epidemic peak. Within such period, 37,011 cases were notified in 2015; 215,327 in 2016; 17,452 in 2017; 8,024 in 2018; 10,768 in 2019, and 3,692 up to the 2020 Epidemiological Week 23^9^ ^-^ ^12^.

The three most studied forms of Zika virus transmission are vectorial transmission by mosquito bites, vertical transmission, and sexual transmission^13^. This manuscript approaches vertical and sexual transmissions (vaginal, anal, and oral sex) due to the CPTG focus. In Brazil, vectorial transmission is the most frequent. It occurs through infected arthropods' bite, with *Aedes aegypti* being the principal vector in this country. It should be highlighted this is the same mosquito that transmits the dengue, chikungunya, and yellow fever viruses^14^. 

Zika virus transplacental transmission brings paramount concern in its scenario due to its potential to cause embryonic and fetal central nervous system structural abnormalities. Several mechanisms are evoked for explaining how the virus reaches the embryo and fetus. As the syncytiotrophoblast is resistant to the Zika virus infection^15^, in early pregnancy, some mechanisms are used by the virus, allowing the penetration in the chorionic villus. One mechanism is the glucosamines cleavage by one of the virus's non-structural proteins (*non-structural*, NS1)^16^. Inside the villus, the Zika virus infects macrophages (Hofbauer cells), where it causes intense proliferation and cellular hyperplasia^17^ ^,^ ^18^. 

Studies have detected Zika virus presence in blood, semen, urine, and saliva, suggesting that it could also be transmitted through these body fluids. However, this kind of transmission is rare^19^ ^-^ ^22^. In places with a high frequency of the vector mosquito, it is difficult to establish the direct transmission risk through sexual intercourse. Therefore, this transmission route may be important in countries where Zika virus infection is not endemic^23^ ^,^ ^25^, although it can also occur in countries with active transmission through arthropods^26^. This transmission mode has already been reported in at least 13 countries without mosquito transmission^27^ ^,^ ^28^.

Zika virus sexual transmission was suggested in 2011 in the United States of America, with the case report of a woman infected with the virus when her partner returned from a trip to Senegal in 2008, when he was infected^23^. Later, Zika virus's RNA presence was reported in semen samples longer than in blood and urine samples in a patient with hematospermia^24^. 

A study conducted in Padova (Italy), from January 2016 to January 2017, showed that Zika virus was detected in the semen of five out of ten tested men, eliminating up to 370 days and a mean clearance of 25 days. However, in general, this viral elimination period is highly variable^28^ ^-^ ^30^. Although viral detection in fluids does not imply new infection viability, sexual transmission from an infected man is facilitated since semen has a higher viral load and longer elimination period than serum and urine^31^. However, there are also cases of sexual transmission from infected women to their sex partners^32^. Zika virus transmission in receptive and active sexual intercourse between men has also been showed^21^ ^,^ ^33^. 

## CLINICAL ASPECTS

**FIGURE 2:**
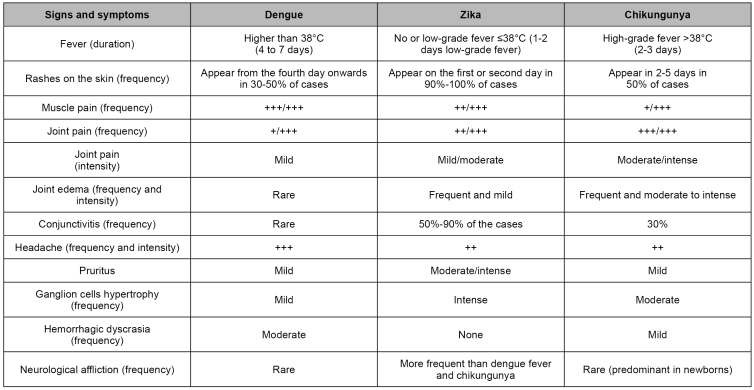
Comparative frequency of most common signs and symptoms in dengue, Zika, and chikungunya infections.

**Source:** adapted from Duarte et al. (2016)^36^, Pernambuco State Health Department (2015)^37^ and Duffy et al. (2009)^39^.

Zika virus infection is an acute, self-limited, feverish disease lasting from three to seven days, usually without severe complications. Most infections are asymptomatic, but from 20% to 25% of infected people have nonspecific clinical manifestations, giving room to the need for differential laboratory diagnosis regarding chikungunya and dengue^34^ ^-^ ^39^. Infection must be suspected in the occurrence of two or more of the symptoms described in Figure 2. The definition of a suspected case is the same for people living in endemic areas and for travelers from those areas and their unprotected sex contacts^25^. 

Among all the possible Zika virus infection complications, those with gestational and neurological outcomes stand out^40^. Even considering the inaccuracies regarding these complications' prevalence, since the real infection incidence is unknown (considering the asymptomatic cases), the causal link between gestational and neurological complications and Zika virus infection has already been scientifically established^41^ ^-^ ^43^. Considering gestational complications, it is estimated that miscarriage rates (1.2% to 3.9 %), eye defects (1.0% to 7.5%), fetal death (0.7% to 1.6%) and malformations (3.1% to 22.8%) are increased due to infection during pregnancy^35^ ^,^ ^44^ ^-^ ^46^. The most feared neurological complication in adults is Guillain-Barré syndrome, whose frequency, estimated by meta-analytical studies, ranges from 0.01% to 1.23%^47^ ^,^ ^48^. 

From the male reproductive point of view, in cases where the virus reaches the testicle, Zika virus aggression to testicular tissues is pointed out, with a prognosis that shall depend on the intensity of the immune and inflammatory response, varying from a simple reversible spermatozoon number reduction to testicular atrophy^49^ ^,^ ^50^. Evaluation conducted among men returning from Zika virus infection endemic areas to a European city without mosquito transmission found macroscopic hematospermia in 9.1%, microscopic hematospermia in 81.8%, and oligospermia in 60% of them^51^. 

## DIAGNOSIS

Laboratory diagnosis during acute Zika virus infection is based on detecting viral RNA through the polymerase chain reaction, mediated by reverse transcription polymerase chain reaction (RT-PCR) in serum or blood up to seven days after symptoms or in urine sample up to 14 days. Although there are cases in which viral RNA can be detected for longer, either in serum or urine and semen, this possibility does not contribute to diagnosing the disease's acute phases^34^. 

In cases of clinical manifestations compatible with Zika virus infection, with a negative result on the RT-PCR diagnostic test, or if more than seven days have elapsed since the onset of symptoms, a serological examination must be requested to identify immunoglobulin M (IgM) for Zika virus and dengue. The IgM can be detected, on average, from the 8^th^ day, and immunoglobulin G (IgG) from the 17^th^ day after the begin of infection. Every serological test must be cautiously interpreted, considering the high number of cross-reactions with other *Flavivirus* ^52^ ^,^ ^53^. 

If the serological result is negative for both viruses, infection by both is dismissed. If either one is positive, the plaque reduction neutralization test must be requested, if available. In cases of the reduction is >10 for dengue virus and <10 for Zika virus, dengue infection is confirmed. If the result is >10 for Zika virus and <10 for dengue virus, Zika virus infection is confirmed. If it is >10 for both viruses, *Flavivirus* infection is established, and if it is <10 for both, both infections are dismissed^34^. 

Due to symptoms overlapping, in regions with high arboviruses prevalence, the differential diagnosis of Zika virus infection must be conducted with all exanthematous diseases, including chikungunya, dengue, and measles. Such differential diagnosis must be expanded in pregnant women, considering syphilis, toxoplasmosis, rubella, cytomegalovirus, and herpes (STORCH), all of which can cause fetal malformations^36^ expanded to Z-STORCH when including malformations caused by congenital Zika virus syndrome.

## TREATMENT

Currently, there is no specific treatment for Zika virus infections. Management implies symptomatic treatment, which includes hydration, analgesics, and antipyretics. Non-steroidal anti-inflammatory drugs must be avoided until dengue diagnosis is dismissed^36^. This principle also applies to pregnant women. For more severe sequelae, such as neurological diseases, medical follow-up must be implemented to assess the adequate treatment to be applied, primarily in specialized centers^40^. 

## SURVEILLANCE, PREVENTION, AND CONTROL

Notification of Zika virus infection suspected cases is mandatory for all Brazilian states. The measure was published in the Brazilian Official Gazette through Ordinance no. 204, of February 17th, 2016. All suspected cases must also be reported to health authorities weekly. For pregnant women with a suspected virus infection or suspected death, notification is immediate, within 24 hours^54^. 

One of the factors reinforcing primary prophylaxis measures' role is based on the lack of specific vaccine or treatment for Zika virus infection and secondary prevention methods for vertical transmission^14^. In primary prophylaxis, *Aedes aegypti* reproduction control and using repellents, and wearing appropriate clothing stand out. It should also be considered that *Aedes aegypti* mosquitoes have preferentially daytime habits and depend on light and higher temperatures^55^. Strategies for biological control of these vectors' population have shown to be promising, such as using *Wolbachia pipiensis* bacteria^56^, which interferes with *Aedes aegypti* reproduction cicle and prevents the eggs laid by infection-free females from hatching when fertilized by a male infected with *Wolbachia* ^57^ ^-^ ^59^. 

Objectively aiming to control the Zika virus sexual dissemination, female or male condoms must be reinforced for possible vectorial or sexual exposure when traveling to high transmission areas and returning to non-endemic areas. The same recommendation should be applied even after the diagnosis of the infection^21^ ^,^ ^30^ ^,^ ^60^ ^,^ ^61^. 

In terms of sex partners, it is essential to reduce transmission between infected people and develop strategies to break the transmission chain through identification and adequate management of sex partners. Risk communication and adequate information for health service users are essential. A gender-specific approach is important, considering that men's and women's prevention responses have shown to be different. Disease awareness is associated with a more preventive and protective attitude by both sexes^62^. 

According to the guidelines by both the American Centers for Disease Control and Prevention and the World Health Organization, the quarantine of protective sexual measures or sex abstinence when returning from travel to endemic regions is of 90 days for men and 60 days for women. These international organizations also share the guideline for protected sex or even sex abstinence for the remainder of the pregnancy when a pregnant woman's partner returns from a Zika virus endemic region or has been diagnosed with the infection^25^ ^,^ ^63^. Brazilian professionals should follow such guidelines until they are incorporated into the next CPTG edition. 

### Zika virus infection during pregnancy

Zika virus vertical transmission, which can occur at any time during pregnancy, has been associated with serious and harmful pregnancy outcomes. Although Zika virus vertical transmission frequency among asymptomatic pregnant women is difficult to measure, there is confirmation of its occurrence^13^. Viral RNA persistence lasts longer among pregnant women, probably due to viral replication in the placenta, increasing fetal exposure, and congenital malformation risk even among asymptomatic mothers^64^ ^-^ ^66^.

Embryonic or fetal central nervous system afflicting spectrum causing malformations and its pathogenesis are not yet fully established. It is known that Zika virus presents neural progenitor cells tropism and that, in the embryonic or embryonic/fetal brain, it stops growth, proliferation, migration, and differentiation of neuronal cells, with severe consequences for neurodevelopment^40^ ^,^ ^44^ ^-^ ^46^ ^,^ ^67^ ^,^ ^68^.

When infection occurs during the first and second trimesters of pregnancy, there is an increased risk of an embryonic or fetal central nervous system structural abnormalities. These infections most often present high variation of anatomical changes, which result in ventriculomegaly, intracranial calcifications, and microcephaly^13^ ^,^ ^14^ ^,^ ^35^ ^,^ ^69^. 

Zika virus infection neonatal changes were grouped under the name of congenital Zika virus syndrome, including microcephaly with the collapse of the skull, thin cerebral cortices with subcortical calcifications, facial disproportion, hypertonia, spasticity, hyperreflexia, seizures, irritability, arthrogryposis, macular scarring, focal pigmentary retinal mottling, blindness, and sensorineural hearing loss^67^ ^,^ ^70^.

**FIGURE 3:**
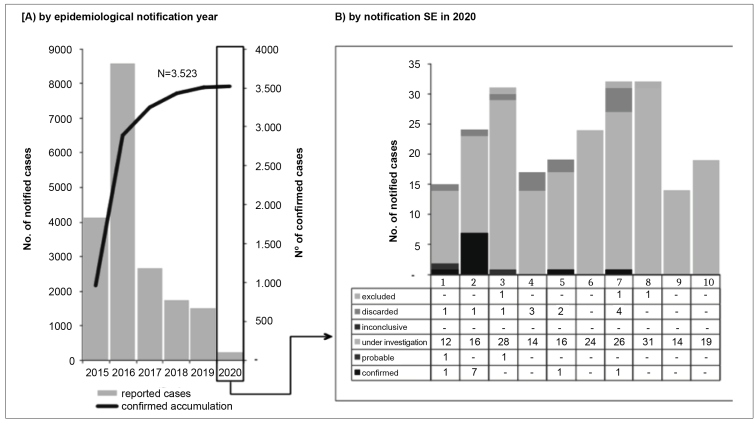
Distribution of notified congenital Zika virus syndrome and other infectious etiologies cases by notification epidemiological year (A) and notification epidemiological week (SE) in 2020 (B), Brazil, 2015 up to SE 10 of 2020.

Source: Health Surveillance Department Epidemiological Bulletin, Ministry of Health. Data updated on March 09th 2020^10^.

The increased microcephaly frequency as causing pregnancy complications in Brazil from 2015 to 2020 was published in the Epidemiological Bulletin of the Health Surveillance Department (Figure 3)^12^. The spinal cord can also be affected, showing architectural distortion, severe neuronal loss, and microcalcifications^64^. However, other changes have also been observed in infections occurring in the third trimester of pregnancy, but detected lately, sometimes after birth, such as eye defects, dysphagia, microcephaly, reduced hearing acuity, electroencephalographic abnormalities, and convulsions, among other health problems^45^. Only the prospection of children exposed to Zika virus in intrauterine life can support a broader understanding of congenital Zika virus syndrome^70^. 

### Low or normal risk prenatal care assistance (pregnant women without Zika virus infection signs)

For pregnant women at low or normal risk, the most critical strategy regarding the Zika virus is to prevent them from getting infected. Reaffirming preventive measures for vector or sexual infection is the most efficient strategy to prevent the spread of the infection^13^ ^,^ ^54^. People who do not live in endemic areas must be advised to avoid traveling to places where Zika virus infection frequency is high^36^ ^,^ ^71^.

From an assistance point of view, after the Zika virus infection epidemic outbreak, the Brazilian Federation of Gynecology and Obstetrics Associations recommends implementing an ultrasound examination around the 12^th^ week, a morphological ultrasound around the 22^nd^ week, and a third examination around the 32^nd^ week^72^ ^,^ ^73^. These tests aim at the early detection of fetal changes resulting from maternal asymptomatic Zika virus infections^13^ ^,^ ^71^. 

Universal screening for prior Zika virus infection among "asymptomatic pregnant women", using serological tests, is not indicated. It is essential to consider the tests' low accuracy, the potential aggregate costs, and the scarce benefits obtained with this measure. Given a positive serology exam showing the presence of antibodies to the virus it is necessary to ensure that a false safety impression cannot be communicated, which could result in reducing the pregnant woman's care regarding other arboviruses (dengue, chikungunya, and yellow fever), diseases that can also present harmful maternal and perinatal outcomes^73^ ^,^ ^74^. 

In cases suggesting Zika virus infection, the pregnant woman must seek medical attention and undergo physical and laboratory examination to guide the diagnosis^75^. The Brazilian Federation of Gynecology and Obstetrics Associations recommends that this pregnancy should be considered a high perinatal risk based on the diagnostic confirmation^73^.

### Prenatal care assistance for pregnant women diagnosed with Zika virus infection

A great demand for psychological support marks the prenatal care of pregnant women diagnosed with Zika virus, which is a fundamental intervention, in addition to compliance with basic prenatal guidelines, such as care associated with blood pressure and weight gain, adequate nutrition, laboratory tests, and vaccines^36^ ^,^ ^73^.

Currently, more than 500 causes are known for microcephaly, in addition to congenital Zika virus infection, with varying severity degrees. Considering that the treatment and pregnancy monitoring assessment, as well as the newborn’s can be influenced by the etiology, the role of conducting diagnostic tests to detect the teratogenic agent is highlighted. In the case of Zika virus infection, the RT-PCR of the amniotic fluid obtained by the amniocentesis may be an alternative for conducting the differential diagnosis^13^ ^,^ ^73^, considering the possibility of false-negative results^62^.

In cases of fetal infection, prenatal service shall require fetal vitality care through cardiotocography and ultrasound. The returning frequency shall depend on each fetal clinical condition, with no requirements to set strict intervals between appointments^13^ ^,^ ^73^. 

### Delivery and breastfeeding

In general, even children affected by congenital Zika virus syndrome can survive labor, considering vaginal delivery as preferable for mothers^71^ ^,^ ^73^. Natural breastfeeding is also regarded as ideal for children born from mothers with Zika virus infection. If there is no contraindication to oral feeding, breastfeeding shall be started^9^. 

### Assisted reproduction

Until now, there have been no reported cases of Zika virus vertical transmission due to pregnancy that have used assisted reproductive techniques, but some care is recommended for couples undergoing infertility treatment. They are: a) presenting negative IgM serology five days before the procedure; b) wait up to 90 days after the appearance of signs and symptoms associated with Zika virus infection when the man was infected or report risky sexual exposure or travel to endemic regions; c) wait up to 60 days after signs/symptoms associated with Zika virus infection when the woman was infected or report risky sexual exposure or travel to regions endemic for this infection^25^ ^,^ ^63^ ^,^ ^75^ ^,^ ^76^. 
